# Multi-omics-based molecular classification of adrenocortical carcinoma predicts response to immunotherapy and targeted treatments

**DOI:** 10.1007/s12672-025-03649-y

**Published:** 2025-10-02

**Authors:** Xingwei Jin, Xianjin Wang, Zhiyuan Wang, Baoxing Huang, Xuejian Zhou, Boke Liu, Yuan Shao, Guoliang Lu

**Affiliations:** 1 Department of UrologyRuijin Hospital, Shanghai Jiao Tong University School of Medicine, Shanghai, 200025 China; 2 Department of Anesthesiology Ruijin Hospital, Shanghai Jiao Tong University School of Medicine, Shanghai, 200025 China

**Keywords:** Adrenocortical carcinoma, Multi-omics integrated analysis, Molecular classification, Targeted therapy

## Abstract

Adrenal cortical carcinoma (ACC) is a rare and highly aggressive malignant tumor with dismal outcomes. Once metastasis occurs, the 5-year survival rate falls below 15%. Current treatment options offer LIMited benefit for advanced disease, Largely due to the absence of well-defined therapeutic targets, and there is an urgent need to develop new molecular classifications to achieve precise treatment strategies. In this study, we integrated multi-omics data including transcriptome, epigenetic, and genomic variation profiles and applied 10 clustering algorithms, identifying two robust molecular subtypes of ACC: Multi-Omics ACC Consensus Subtyping (MACCS)1 and MACCS2. Biologically, MACCS1 exhibits a proliferation-driven phenotype, whereas MACCS2 displays an immune activation state. Drug sensitivity analysis further revealed that MACCS2 tumors were more responsive to immune checkpoint inhibitors, while MACCS1 showed sensitivity to antiangiogenic tyrosine kinase inhibition. Using a random forest algorithm, we identified HOXC11 as a key prognostic factor within MACCS1, with high expression associated with tumor progression. Functional assays confirmed that silencing HOXC11 significantly reduced the proliferation of ACC cells. Survival analysis showed that the prognosis of patients with MACCS1 had markedly worse outcomes compared to those with MACCS2. Collectively, this study provides a theoretical basis for the molecular classification of ACC and personalized precision treatment, such as immunotherapy and targeted therapy, and highlight HOXC11 as a potential therapeutic target.

**Supplementary Information:**

The online version contains supplementary material available at 10.1007/s12672-025-03649-y.

## Introduction

Adrenal cortical carcinoma (ACC) is a rare but highly lethal endocrine malignancy [1, 2]. It is characterized by aggressive growth and early metastasis, leading to poor outcomes [3]. Nearly 75% of patients develop distant metastases, and the 5-year survival rate remains below 15%, creating a substantial health and economic burden worldwide [4]. Because ACC often presents with nonspecific symptoms, most patients are diagnosed at advanced stages. Radical surgery with adjuvant mitotane remains the standard treatment, while tumor heterogeneity and poorly understood resistance mechanisms limit therapeutic efficacy [5–8]. Therefore, establishing refined molecular subtypes and identifying novel therapeutic targets are essential steps toward improving the clinical management and extending the survival of patients with ACC [9–11].

Despite growing interest, immunotherapy has shown limited benefit in ACC. The Tumor microenvironment (TME) of ACC is typically characterized by an “immune desert” phenotype, with markedly reduced infiltration of effector immune cells such as CD8⁺ T cells and dendritic cells compared to immunologically “hot” tumors like melanoma and lung cancer, which hinders the initiation and effect of anti-tumor immune response [12, 13]. Several targeted and immune-based therapies, including lenvatinib, cabozantinib, and pembrolizumab, have been investigated [14, 15]. Among them, Lenvatinib has demonstrated a disease control rate of approximately 60% [16, 17], but the duration of response is short, and there is an absence of reliable biomarkers for predicting treatment efficacy [18]. Given the rarity of ACC and the challenges of single-modality approaches, integrating multi-omics profiling with advanced machine learning provides an opportunity to uncover new therapeutic targets and predictive biomarkers [19–21].

Previous efforts at ACC classification have predominantly relied on transcriptomic clustering or single-omics analyses, which yielded prognostic insights but limited therapeutic implications [22]. To address this gap, we conducted an integrative analysis of transcriptomic, epigenetic, and genomic variation data to refine ACC molecular subtypes and assess their clinical relevance. By applying multiple clustering algorithms and validating with cluster prediction index (CPI) and Gap statistics (a statistical method for determining the number of clusters), we identified two robust subtypes with reproducible biological distinctions across omics layers [23]. These subtypes exhibited differential proliferative activity, immune contexture, and metabolic states, providing clinically meaningful insights into tumor behavior and treatment response. On this basis, we propose the Multi-Omics ACC Consensus Subtyping (MACCS) model, which bridges the gap between prognostic classification and actionable clinical decision-making by linking molecular subtype identity with both biological mechanisms and therapeutic vulnerabilities.

## Materials and methods

### Dataset collection

Multi-omics data of ACC patients were obtained from The Cancer Genome Atlas (TCGA, https://portal.gdc.cancer.gov/) [24], including transcriptome profiles, DNA methylation, somatic mutations, and corresponding survival information. Only patients with complete multi-omics and prognostic data were included in the analysis. For external validation, four independent ACC gene expression datasets were retrieved from the Gene Expression Omnibus (GEO) database: GSE10927 (*n* = 24), GSE33371 (*n* = 23), GSE70621 (*n* = 29), and GSE19750 (*n* = 22). As all data were derived from public repositories, no additional ethics approval was required.

### Identification and validation of multi-omics molecular typing

To ensure stable and reliable molecular subtyping, we applied a multi-omics consensus analysis strategy. Prognosis-associated features were first identified within each omics layer using univariate Cox regression. These features were then input into the R package MOVICS (version 1.0) [25], which integrates multiple clustering algorithms and provides outputs on subtype characteristics, prognostic differences, and therapeutic sensitivity. We used 10 clustering algorithms from MOVICS (COCA, Consensus Clustering, CLML, IntNMF, iClusterBayes, PINSPlus, NEMO, MoCluster, LRAcluster, and SNF) to cluster prognostic features. We perform consensus analysis on the clustering results of each algorithm using combined classification of the consensus set to obtain clustering results with high robustness.

The optimal number of clusters was determined by combining prior biological knowledge with two quantitative indices: the Cluster Prediction Index (CPI) and Gap statistics [26, 27]. The number that resulted in the maximum value of gap statistics and CPI was selected as the optimal number of clusters for the input data. To evaluate the robustness and reproducibility of the multi-omic subtypes identified from the TCGA-ACCs dataset, we performed nearest template prediction (NTP, a supervised classification approach that assigns samples to predefined classes based on correlation to class templates) analysis using the runNTP function in the MOVICS package.

### GO, KEGG, and GSVA analysis

In this study, the DESeq2 software package [28] was used to calculate the differentially expressed genes (DEGs) between MACCS1 and MACCS2, with the application parameters of |log2FC| >1 and *p* < 0.05. To better understand the biological differences between the two groups, the DEGs were subjected to Gene Ontology (GO) and Kyoto Encyclopedia of Genes and Genomes (KEGG) pathway functional enrichment analysis using the clusterProfiler package (version 4.14.6) in R language. GO analysis included three classification levels: cellular component (CC), biological process (BP), and molecular function (MF), and the top 10 terms in each category were displayed to analyze the biological characteristics of each molecular subtype. KEGG pathways can be used to identify potential signaling pathways. We used the GSVA package to perform single-sample gene set enrichment analysis (ssGSEA) [29], and the limma software package [30] to analyze the differences in pathway scores between subgroups. The results of all biological pathway enrichment analyses were visualized using the ggplot2 toolkit (version 3.4.0), and a *p* < 0.05 value for enriched words was considered significantly enriched. The gene sets included in our analysis are all from the built-in gene sets in MSigDB, IOBR, and MOVICS software packages.

### Immune infiltration analysis and genomic analysis

To investigate the differences in responses to immunotherapy between the two groups, we used multiple algorithms to compare the differences in immune components between the two groups, including immune cells, expression levels of immune checkpoint-related molecules, and immune scores. We studied the infiltration of individual immune cells in the tumor by ssGSEA and calculated the built-in immune-related gene scores of IOBR and MOVICS in each sample; The maftools package [31] was used to calculate the TMB of each sample and visualize the mutation map. Finally, the sensitivity of different subgroups to immunotherapy was compared by using the TIDE algorithm [32].

### Drug sensitivity analysis

This study analyzed the sensitivity of different subgroups of patients to various drugs by using the drug sensitivity data of tumor cell lines in the Genomics of Drug Sensitivity in Cancer (GDSC, a public resource linking genetic features of cancer cell lines to drug response profiles) database (https://www.cancerrxgene.org/) [33]. The half-maximal inhibitory concentration (IC_50_) of each sample was calculated using pRRophetic in R language. The lower the IC_50_ value, the more sensitive the sample is to the drug. In addition, we calculated potential treatment-sensitive targets in poor prognosis subgroups based on the Connectivity Map (cMap) database [34].

### In vitro experiment

The ACC cell line NCI-H295R [35] used in the in vitro experiments of this study was obtained from the Cell Bank of the Chinese Academy of Sciences (Shanghai, China). The cells were cultured in Dulbecco’s Modified Eagle’s Medium (DMEM) supplemented with 10% fetal bovine serum (FBS) and 1% penicillin-streptomycin at 37 °C and 5% humidity. Cells were routinely passaged every 2–3 days, and all experiments were performed during the logarithmic growth phase. NCI-H295R cells were selected because they retain multiple steroidogenic enzyme activities across adrenal cortex zones, aligning them with MACCS1’s proliferative and steroidogenic features [36]. The effect of HOXC11 interference on ACC cell proliferation was verified using a cell counting kit, and the cell experiment was evaluated according to the instructions, and the absorbance was measured at a wavelength of 450 nm using a microplate reader.

### Statistical test

All data processing, statistical tests, and visualizations in this study were performed using R language (version 4.5.0) and SPSS software (version 29.0). The Wilcoxon rank sum test or t test was used to compare the differences in continuous variables between the two groups. Cox regression and Kaplan-Meier analysis were used for survival analysis. A two-tailed test was used, and a P value < 0.05 was set as statistically significant. No specific code was generated in this study, and all analyses were completed using the default parameters of the corresponding software and R package.

## Results

### Identification of novel ACC molecular subtypes based on multi-omics consensus clustering

**Fig. 1:**
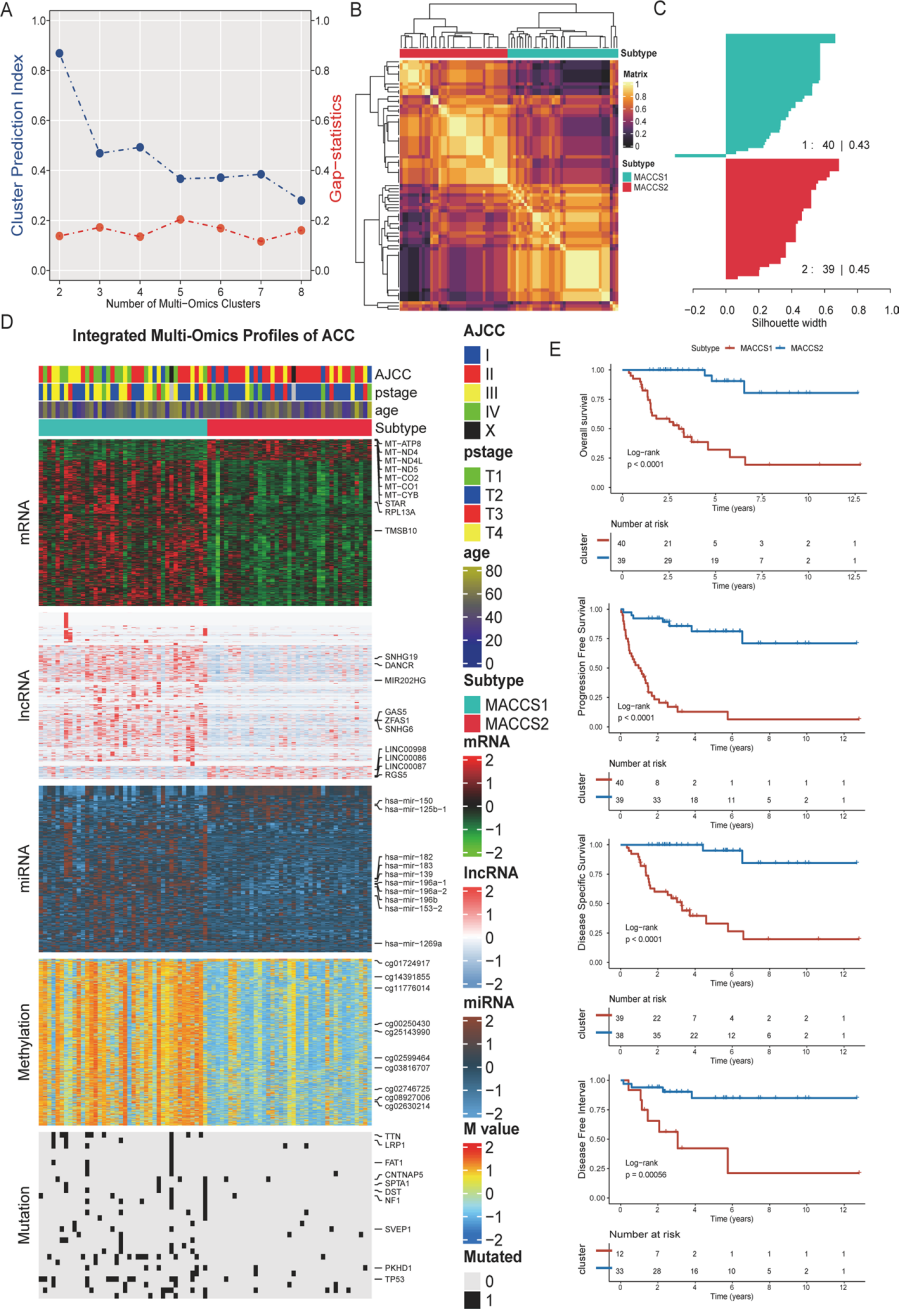
The landscape of multiomics differences between MACCS1 and MACCS2. **A** Clustering prediction index and gap statistics indicating the optimal cluster number for TCGA-ACC; **B** Consensus matrix for two subgroups based on the 10 algorithms; **C** Silhouette plot showing consistency between subgroups; **D**, **E** Kaplan‒Meier survival curves of OS and DFI for MACCS1 and MACCS2

To systematically characterize the molecular heterogeneity of ACC and construct a refined molecular classification system, we integrated transcriptomic (mRNA, lncRNA, miRNA), DNA methylation, and somatic mutation data from the TCGA and GEO datasets. Using ten multi-omics clustering algorithms implemented in the MOVICS framework, we performed unsupervised consensus clustering. Both the CPI and Gap Statistics consistently indicated that 2 clusters represented the optimal solution (Fig. 1A). The concordance between the consensus matrix and the subtype profiles further confirmed the robustness of the classification, leading us to define two novel molecular subtypes, designated MACCS1 and MACCS2 (multi-omics based ACC subtype 1 and 2; Fig. 1B, C).

We next examined subtype-specific molecular features across omics Layers. The top 10 differential features from each layer were integrated into a comprehensive heatmap (Fig. 1D). At the mRNA level, MACCS1 tumors showed elevated expression of SNHG19, MIR202HG, and GAS5, genes implicated in cell migration and immune evasion, and associated with unfavorable prognosis. By contrast, MACCS2 tumors exhibited enrichment of mitochondrial genes such as MT-ATP8, MT-ND4, and MT-ND4L, suggesting enhanced oxidative phosphorylation, metabolic reprogramming, and potential resistance to stress or metastatic adaptation. At the miRNA level, MACCS1 tumors were enriched for miR-183, miR-139, and miR-196b, which likely exert post-transcriptional regulation of oncogenic pathways by targeting cell cycle and mRNA stability. DNA methylation analysis revealed subtype-specific CpG modifications (*cg01724917* and *cg11776014*), indicating an important role for epigenetic mechanisms in subtype formation. Mutational profiling showed that MACCS1 harbored higher mutation frequencies in key tumor suppressors such as *TP53* and *CNTNAP5*, consistent with greater genomic instability and disease aggressiveness. Clinically, survival analysis demonstrated that patients with MACCS1 had significantly worse overall survival (OS), progression-free survival (PFS), disease-specific survival (DSS), and disease-free interval (DFI) compared with MACCS2 (Fig. 1E), underscoring MACCS1 as a high-risk subtype with poor prognosis.

**Fig. 2:**
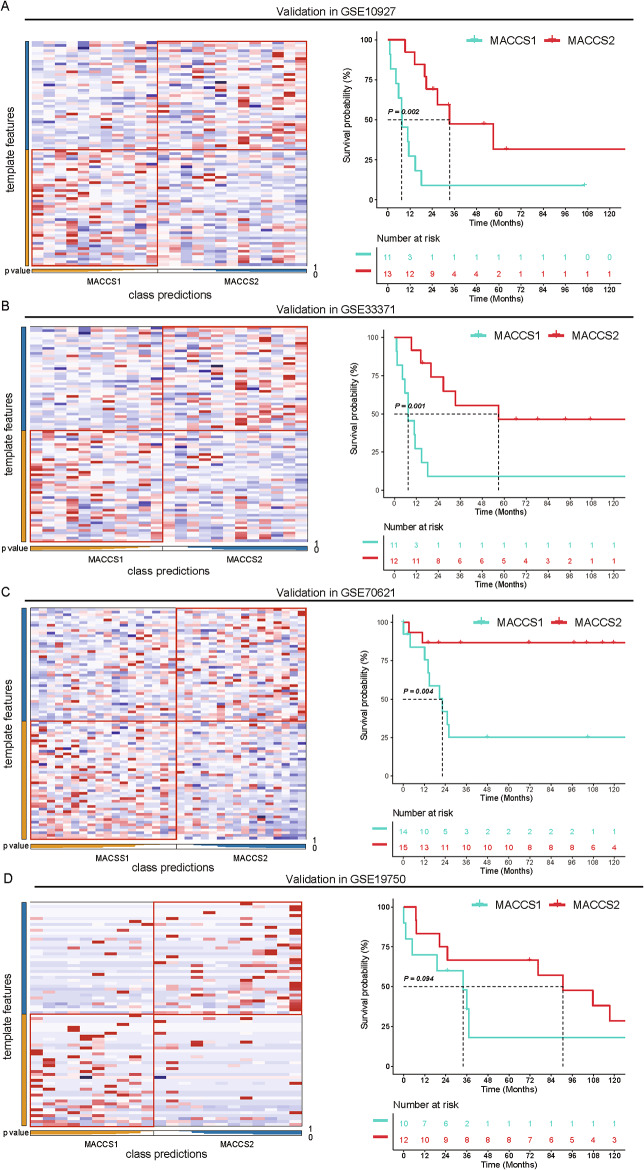
Validation of the robustness of MACCS1 and MACCS2 in external data using the NTP algorithm. **A** Representation of sarcoma classification in the GSE17674 cohort (left) and assessment of overall survival (right); and **B** GSE33371; **C** GSE70621; and **D** GSE19750

To validate the robustness and reproducibility of this classification, we performed NTP algorithm across four independent GEO datasets (GSE10927, GSE33371, GSE7062, and GSE19750). In each dataset, patients were reliably assigned to MACCS1 or MACCS2, and the heatmaps of expression patterns closely recapitulated the subtype signatures observed in TCGA (Fig. 2A and D). Survival analysis showed that patients with MACCS1 subtype had markedly inferior outcomes compared with those with MACCS2 subtype. Collectively, these findings demonstrate that MACCS1 and MACCS2 represent reproducible and clinically meaningful ACC subtypes, highlighting their potential utility for patient stratification and prognostic assessment.

### MACCS classification is associated with proliferation behavior in ACC

**Fig. 3:**
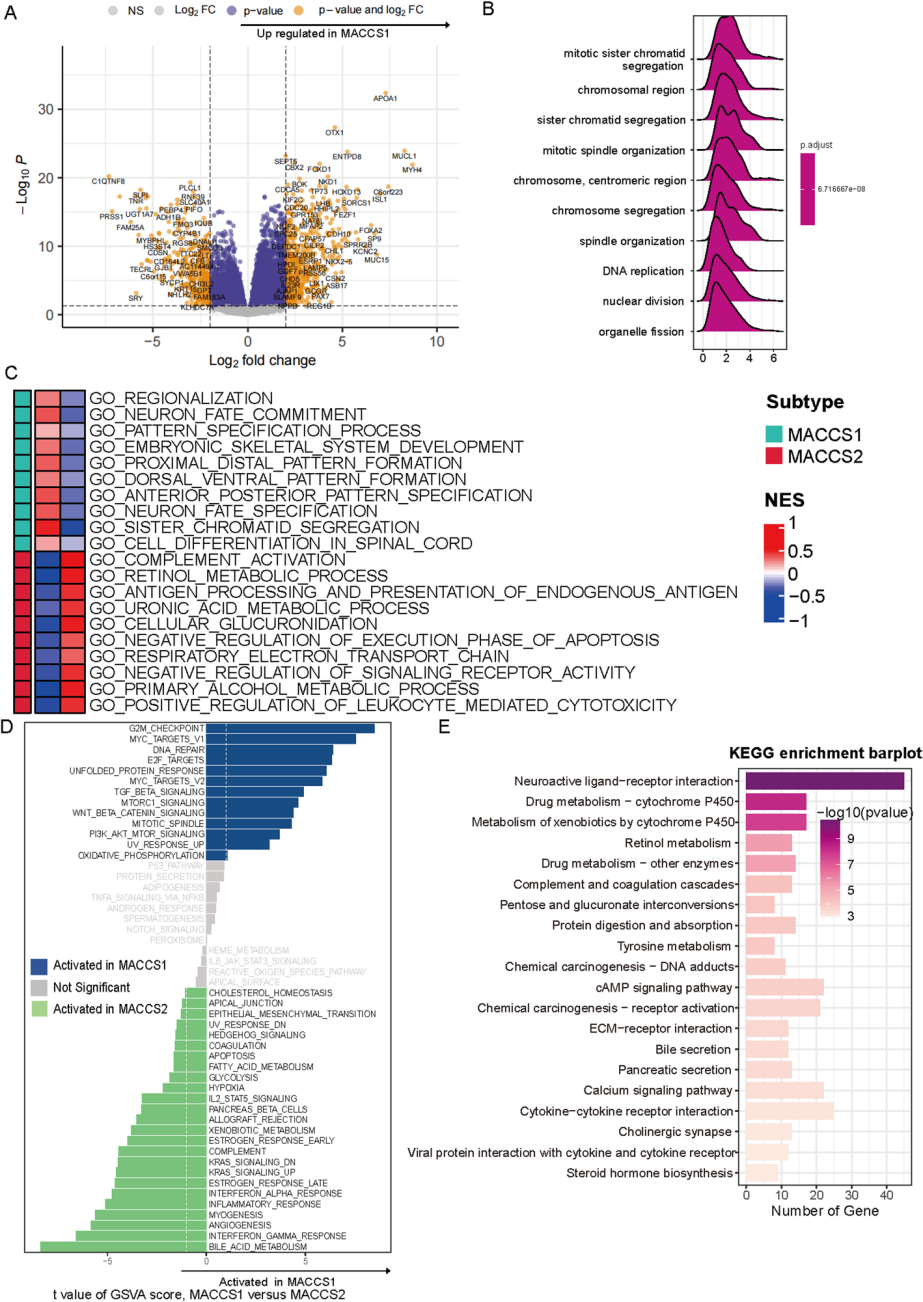
Biological processes and pathways activated in MACCS1. **A** Differential expressed genes between the two groups; **B** GO terms including of DEGs between MACCS1 and MACCS2; **B** Ridge plot showing enriched KEGG pathways in MACCS1 based on Reactome database; **C** Heatmap showing enriched pathways from MOVICS package between groups; **D** Bar plot indicating different enrichment scores of hallmarks; **E** KEGG pathways between groups

To explore biological differences between the MACCS subtypes, we conducted differential expression and enrichment analyses in the TCGA-ACC cohort. GO analysis showed that in the biological processes (BP) category, genes such as *APOA1* and *ENTPD8* were upregulated in MACCS1, suggesting a role for metabolic reprogramming in fueling tumor invasion and metastasis. Elevated expression of genes including *HOXD13* and *OTX1* further indicated that MACCS1 may promote neuron-like migratory properties, thereby facilitating neural and distant metastasis (Fig. 3A). Functional annotation revealed that differentially expressed genes in MACCS1 were enriched in cell-cycle related pathways such as chromatid segregation, DNA replication, and organelle division (Fig. 3B). Consistently, MOVICS-based gene set analysis highlighted enrichment in developmental and differentiation processes, including tissue regionalization, aligning with the aggressive phenotype of this subtype (Fig. 3C). GSVA analysis confirmed that MACCS1 was associated with activation of canonical oncogenic pathways, including E2F targets, G2/M checkpoint, and PI3K/AKT/mTOR signaling. In contrast, MACCS2 was enriched in tumor-suppressive and immune-associated pathways such as KRAS signaling, interferon-γ response, and angiogenesis (Fig. 3D), underscoring the functional divergence between the two subtypes. KEGG pathway analysis further demonstrated that MACCS1-specific genes were concentrated in pathways linked to complement and coagulation cascades and drug metabolism, suggesting involvement in immune regulation, and metabolic homeostasis (Fig. 3E).

To validate these phenotypic differences, we further annotated MACCS2 with IOBR-derived gene sets. Metabolic pathways, including homocysteine biosynthesis, methionine cycle, purine/pyrimidine metabolism, and folate one-carbon metabolism, were significantly downregulated (Figure S1A). In contrast, immune-related pathways, including immune cell proliferation and infiltration signatures, were markedly upregulated, consistent with MACCS2 representing an “immune-hot” phenotype associated with more favorable prognosis (Figure S1B).

### Immune landscape profiling reveals MACCS2 as an immune-hot, checkpoint-responsive subtype in ACC

**Fig. 4:**
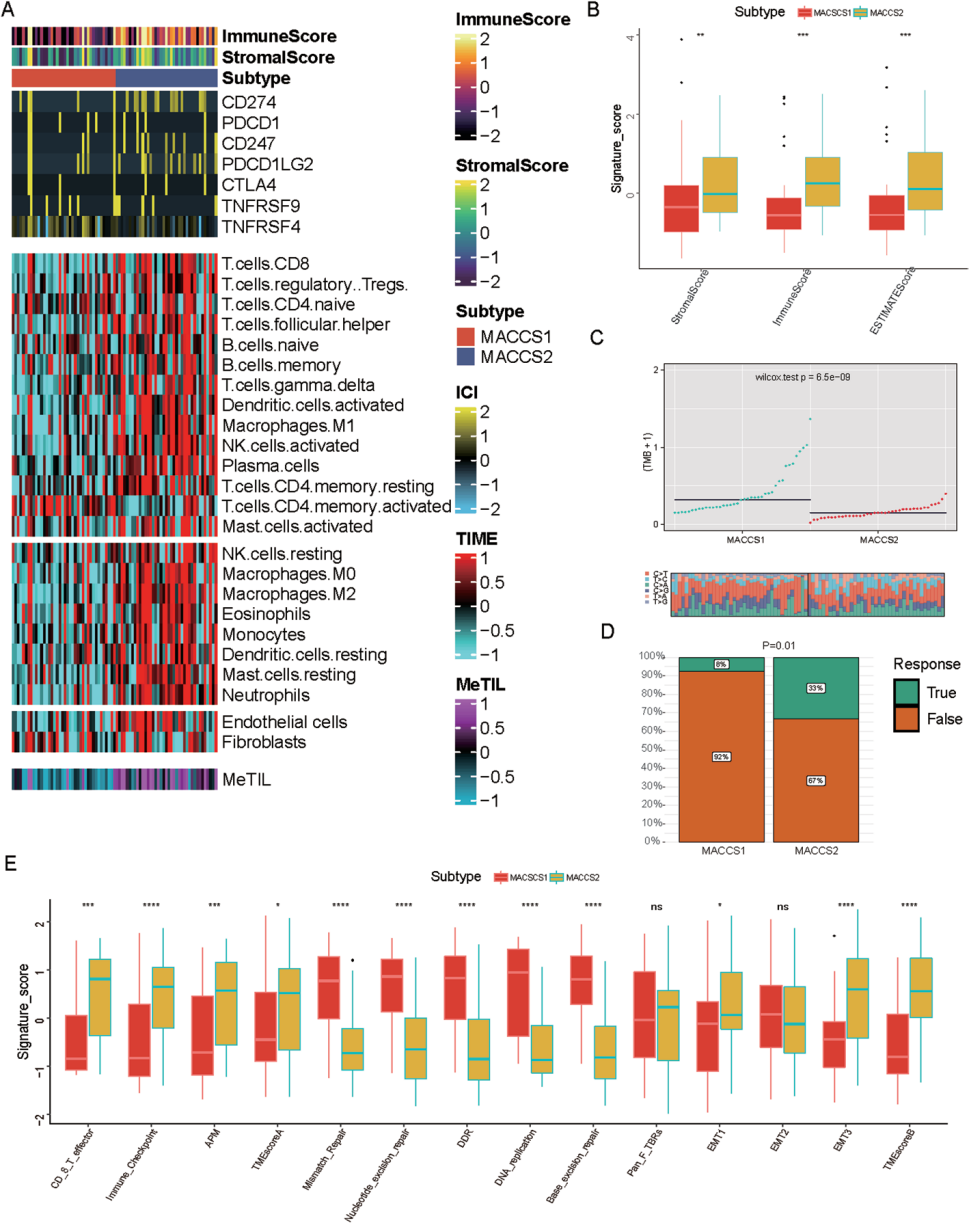
MACCS2 exhibits an immune-hot phenotype. **A** Heatmap showing completely different immune landscapes of MACCS1 and MACCS2; **B** Immune dysfunction scores between groups; **C** Comparison of MACCS11 and MACCS2 Tumor Mutation Burden (TMB) levels; **D** Response rates of MACCS1 and MACCS2 to immunotherapy; **E** Box plot indicating different enrichment scores of anti-tumor-related immunity scores between MACCS11 and MACCS2

To investigate the immunological basis underlying differential therapeutic sensitivity of the MACCS subtypes, we compared immune-related gene expression, immune cell infiltration, and predictive markers of immunotherapy response between MACCS1 and MACCS2. Key immune checkpoint molecules, including *CD274* (*PD-L1*), *CD247*, and *PDCD1LG2* (*PD-L2*), were significantly upregulated in MACCS2, suggesting heightened sensitivity to immune checkpoint inhibitors (ICIs). Consistently, infiltration of multiple immune cell populations including activated CD4 memory T cells was markedly higher in MACCS2, forming an “immune-hot” TME (Fig. 4A). ESTIMATE analysis further confirmed significantly higher immune, stromal, and composite scores in MACCS2 compared with MACCS1 (Fig. 4B). Interestingly, although MACCS1 displayed higher tumor mutational burden (TMB) and was theoretically more immunogenic (Fig. 4C), it lacked substantial immune activation, indicating the likely presence of immune evasion mechanisms.

To assess predicted response to immunotherapy, we applied the TIDE algorithm. Results indicated that MACCS2 patients were more likely to respond to ICIs, consistent with their higher expression of immune checkpoint genes (Fig. 4D). Pathway enrichment analysis revealed that MACCS2 was strongly associated with enhanced CD8⁺ T cell activity, antigen presentation, and TME activation, whereas MACCS1 showed enrichment in pathways related to epithelial–mesenchymal transition (EMT) and DNA repair (MMR, NER, BER), which may promote immune escape by stabilizing the genome and reinforcing immunosuppressive signaling (Fig. 4E).

Collectively, these findings identify MACCS2 as an immune-hot subtype with a favorable immunotherapy profile, suggesting that ICI-based regimens may represent an effective strategy for this group. By contrast, the immunosuppressive phenotype of MACCS1, despite its high TMB, may render it more suitable for targeted agents or rational combination therapies.

### Subtype-specific drug sensitivity profiling identifies candidate therapeutics for MACCS1 and MACCS2 in ACC

**Fig. 5:**
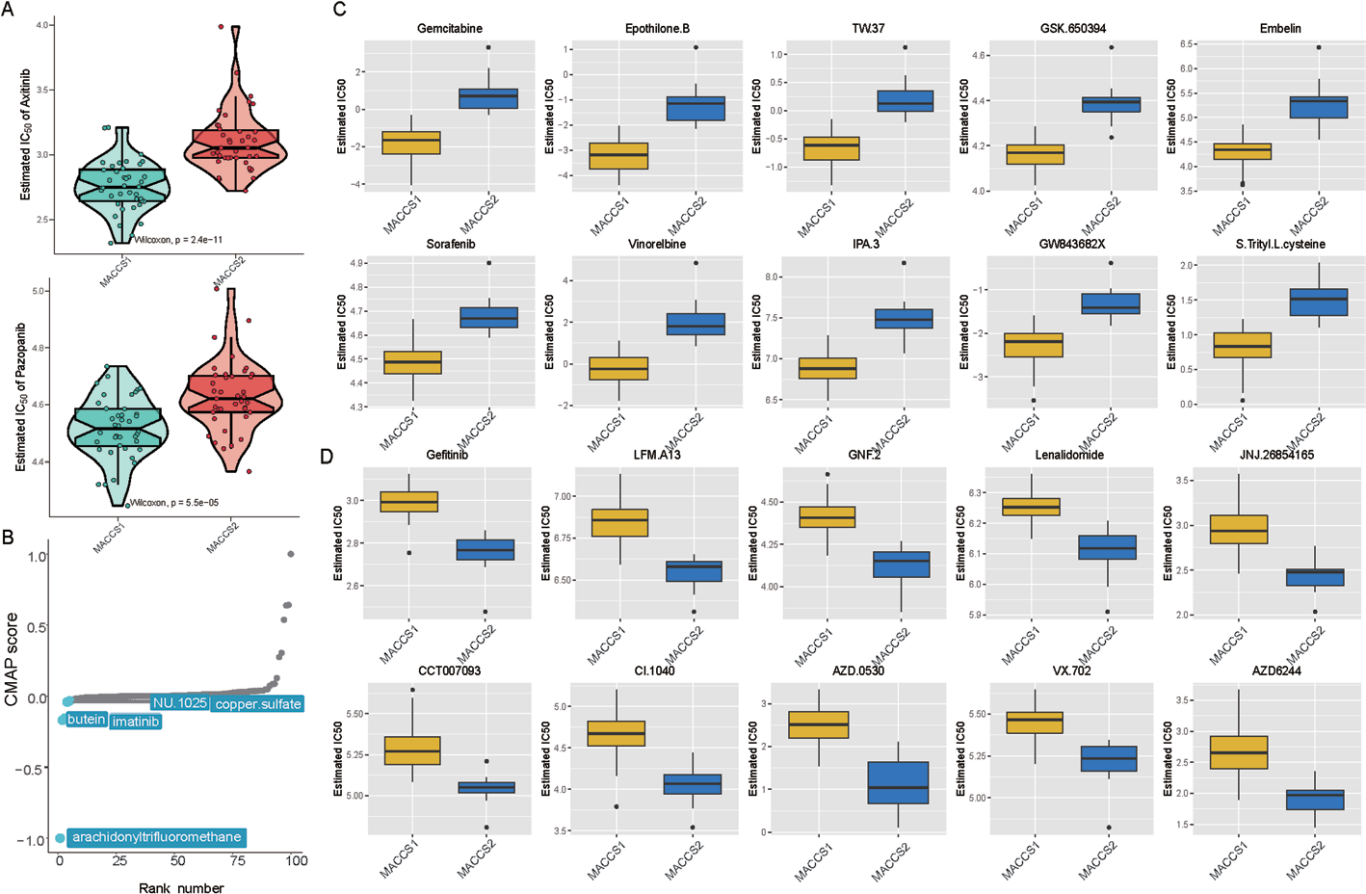
Identification of potential therapeutic drugs for different groups. **A** Different sensitivity to target agents between MACCS1 and MACCS2; **B** Potential agents effective for MACCS1 based on cMap database; **C** Predicted IC50 suitable for MACCS1 and (**D**) MACCS2 from the GDSC database

To explore therapeutic vulnerabilities of the ACC molecular subtypes, we compared predicted drug sensitivities between MACCS1 and MACCS2. Using the MOVICS package, we found that MACCS1 tumors exhibited greater sensitivity to the tyrosine kinase inhibitors axitinib and pazopanib (Fig. 5A). Integration with CMAP database analysis further identified candidate small molecules with potential efficacy in MACCS1, including arachidonoyl trifluoromethane, butein, imatinib, NU-1025, and copper sulfate (Fig. 5B). These compounds are predicted to exert antitumor effects through inhibition of the AKT/mTOR pathway and induction of apoptosis or oxidative stress–related autophagy.

We next applied the GDSC resource to predict IC50 values across subtypes. In MACCS1, predicted sensitivity was observed for multiple agents such as gemcitabine, epothilone-B, TW-37, GSK-650,394, embelin, sorafenib, vinorelbine, IPA-3, GW843682X, and S-trityl-L-cysteine, supporting this subtype’s susceptibility to cytotoxic drugs and kinase inhibitors (Fig. 5C). Conversely, MACCS2 displayed higher predicted sensitivity to a distinct set of targeted compounds, including gefitinib, LFM-A13, GNF-2, lenalidomide, JNJ-26,854,165, CCT007093, CI-1040, AZD-0530, VX-702, and AZD6244 (Fig. 5D). Most of these agents act as inhibitors of the EGFR, MAPK, and JAK/STAT pathways, suggesting that MACCS2 tumors may be particularly dependent on these signaling cascades.

### MACCS1 and MACCS2 exhibit distinct genomic profiles

**Fig. 6:**
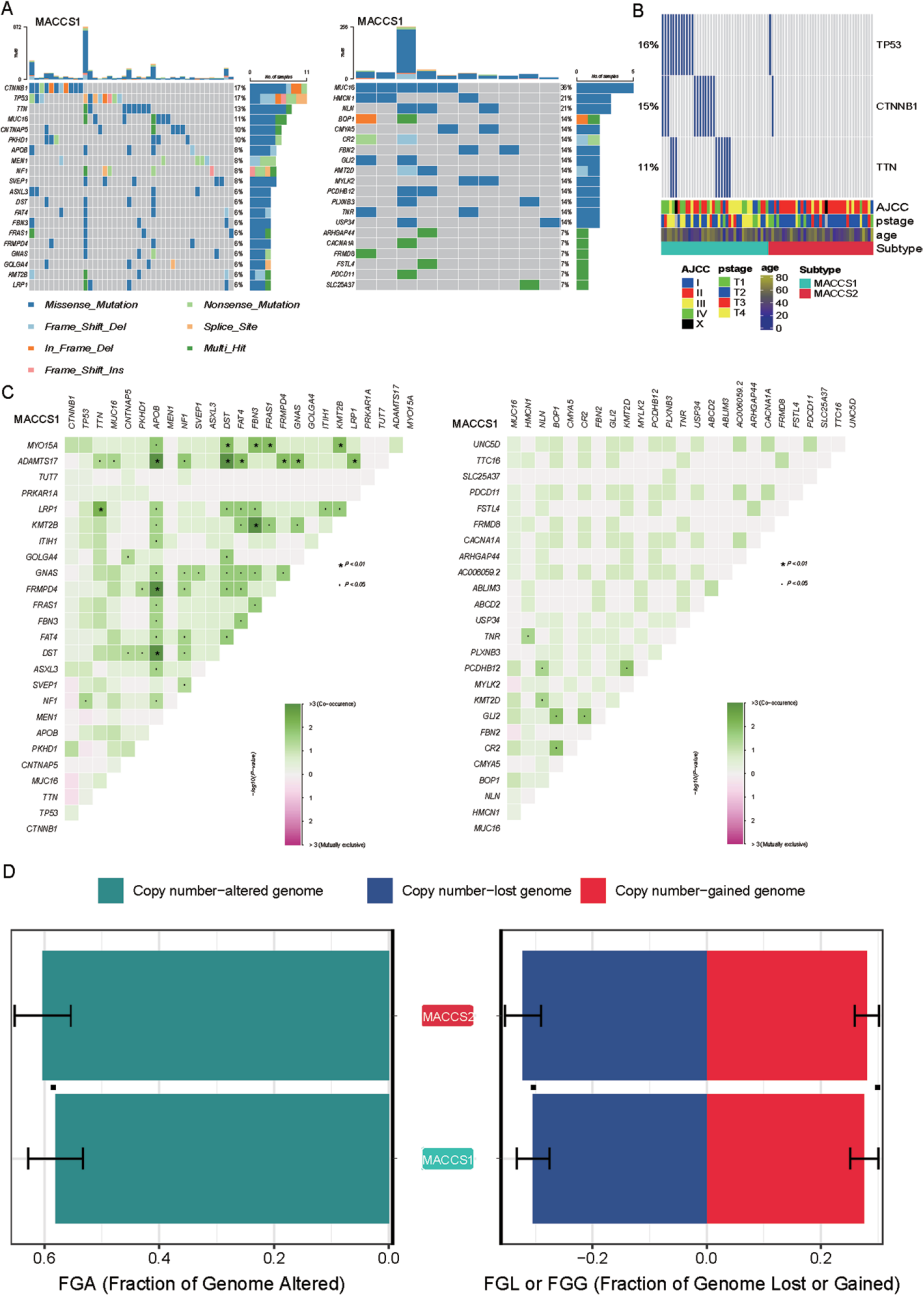
Landscapes of mutation patterns between subtypes. **A** The waterfall plot shows the situation of high-frequency mutated genes in MACCS1 and MACCS2; **B** The characteristic mutant genes of MACCS1 and MACCS2 and their relationships with AJCC and pathological staging; **C** The co -mutation frequency of MACCS1 and MACCS2 subtypes; **D** Differences in copy number variations between MACCS1 and MACCS2

To delineate the genomic characteristics of the MACCS subtypes, we compared their mutational spectra, co-mutation networks, and copy number variation (CNV) patterns. In MACCS1, the five most frequently mutated genes were *CTNNB1* (17%), *TP53* (17%), *TTN* (13%), *MUC16* (11%), and *CNTNAP5* (10%) (Fig. 6A). By contrast, the mutational profile of MACCS2 was dominated by *MUC16* (36%), followed by *HMCN1* (21%), *NLN* (21%), *BOP1* (14%), and *CMYA5* (14%) (Fig. 6A). Most of these alterations involve structural or metabolic genes and are likely “passenger” mutations with limited translational relevance, except for BOP1, which has been implicated in oncogenic processes. Notably, TP53, CTNNB1, and TTN mutations were enriched in MACCS1 and correlated with higher AJCC and pathological stages (Fig. 6B).

Co-mutation analysis revealed that MACCS1 exhibited a more complex mutational coordination pattern, with significant co-occurring pairs such as MYO15A–DST, MYO15A–FNBP3, and MYO15A–FRAS1, implying dysregulation of highly interconnected signaling pathways. In contrast, MACCS2 showed fewer significant co-mutation relationships (Fig. 6C). Analysis of CNVs showed no significant difference in the overall burden of genomic alterations (FGA) between subtypes. Likewise, the frequencies of copy number gains (FGG) and losses (FGL) were comparable between MACCS1 and MACCS2, indicating that CNV is unlikely to be the major determinant of subtype divergence (Fig. 6D).

In summary, MACCS1 is characterized by enrichment of canonical driver mutations and complex co-mutation networks consistent with genomic instability, whereas MACCS2 harbors a higher frequency of largely non-driver mutations with relatively mild genomic disruption.

### HOXC11 is a potential diagnostic and therapeutic target for ACC patients

**Fig. 7:**
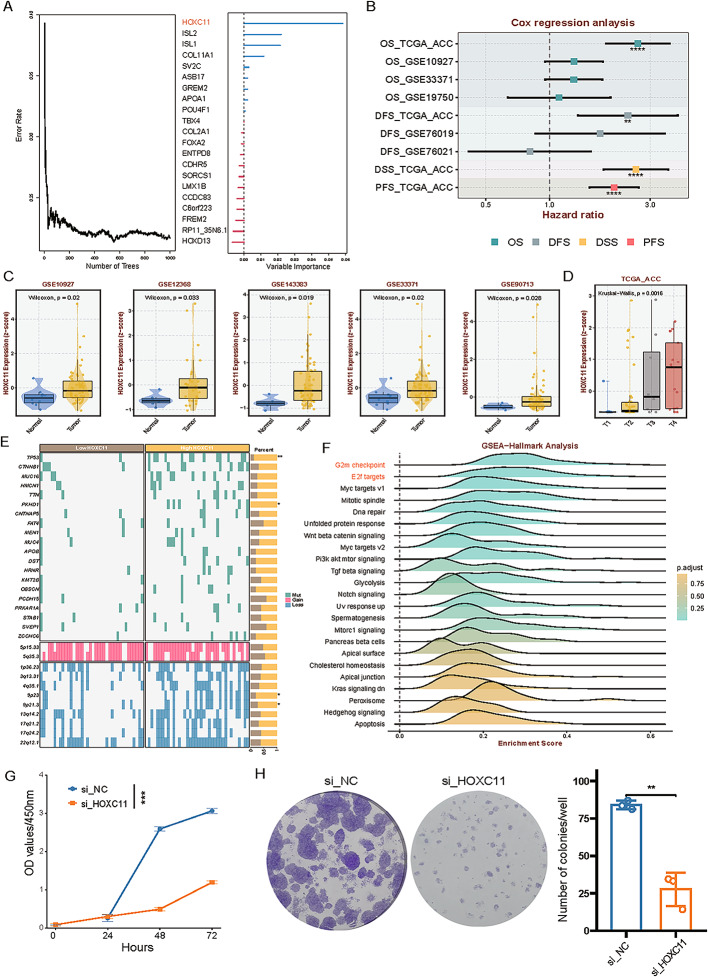
The prognostic and prognostic role of HOXC11 in ACC. **A** Random forest tree ranking the prognostic importance of example genes in the MACCS1 group; **B** Univariate analysis of the prognostic role of HOXC11 in different ACC cohorts; **C** Expression differences and (**D**) prognostic role of HOXC11 in ACC; **E** SNV and CNV differences between HOXC11 low and high expression groups; **F** GO terms of HOXC11 based on correlation analysis in TCGA-ACC; **G** CCK8 assay and **H** clone formation assessing the impact of HOXC11 on ACC proliferation

Given the markedly poor prognosis of the MACCS1 subtype, we applied random forest and univariate Cox regression analyses to prioritize subtype-specific prognostic genes. *HOXC11* emerged as the top-ranked candidate with the highest prognostic importance score, identifying it as a key molecular marker of MACCS1 (Fig. 7A). In addition, across multiple datasets, high *HOXC11* expression was significantly associated with worse OS, DFS, DSS, and PFS (HR > 1, *P* < 0.05, Fig. 7B). Moreover, *HOXC11* expression was markedly elevated in ACC tumor tissues compared with adjacent normal tissues (Fig. 7C), and was significantly higher in advanced-stage tumors (T3/T4) than in early-stage tumors (T1/T2) (Fig. 7D), implicating its role in disease progression.

To investigate the genomic basis of aberrant *HOXC11* expression, we stratified TCGA-ACC samples into high- and low-expression groups using the median *HOXC11* level as the cutoff. The high-expression group displayed increased mutation frequencies in *TP53* and *PKHD1*, along with higher frequencies of deletion events at 9p23 and 9p21.3, suggesting disruption of tumor suppressor pathways (Fig. 7E). Over-representation analysis further demonstrated enrichment of cell cycle–related signaling pathways, including the G2/M checkpoint and E2F targets, in the *HOXC11*-high group (Fig. 7F), supporting its role as a driver of proliferative signaling in ACC.

Functional validation was performed in vitro. Silencing *HOXC11* expression significantly reduced tumor cell proliferation (Fig. 7G) and impaired colony-forming capacity (Fig. 7H), confirming that *HOXC11* promotes the proliferative phenotype of ACC cells. Collectively, these findings highlight *HOXC11* as a potential diagnostic biomarker and therapeutic target in ACC, particularly in patients with the high-risk MACCS1 subtype.

## Discussion

ACC is a rare but highly aggressive malignancy that is often diagnosed at advanced or metastatic stages, which severely limits treatment effectiveness [37]. Its invasion and metastasis process involves the activation of multiple pro-migratory pathways by tumor-intrinsic gene mutations (such as TP53 and CTNNB1), as well as immunosuppression and angiogenesis induced by TME remodeling [38, 39]. While traditional therapies benefit early-stage patients, there remain no clearly defined molecular targets or effective drugs for advanced ACC, underscoring the urgent need for novel therapeutic strategies [40, 41].

In this study, we integrated transcriptomic, epigenetic, and genomic data to establish two robust ACC molecular subtypes: MACCS1, characterized by proliferative and poor-prognosis features, and MACCS2, enriched in metabolic and immune activity. This classification was consistently validated across multiple independent cohorts and not only achieved statistical robustness but also reflected biologically coherent and clinically relevant distinctions. MACCS1 was dominated by cell cycle and DNA repair programs, while MACCS2 exhibited active immune engagement. Compared with prior ACC classifications that relied on single-omics or transcriptomic clustering, our MACCS framework incorporates multiple molecular layers and provides clearer functional separation. Importantly, MACCS subtypes are linked to both prognosis and therapeutic response, enhancing their potential clinical utility.

In MACCS1, the five most frequently mutated genes were *CTNNB1*, *TP53*, TTN, *MUC16*, and *CNTNAP5*. By contrast, the mutational profile of MACCS2 was dominated by *MUC16* and *CMYA5*. Although *MUC16* or *CMYA5* are not classic ACC drivers, their recurrent mutations in MACCS2 suggest functional contributions to tumor biology. *MUC16* has been implicated in modulating cell adhesion, immune evasion, and chemoresistance in other cancers [42], while CMYA5 is involved in cytoskeletal organization and could affect cell motility [43]. Their recurrent mutations in MACCS2 suggest possible contributions to TME interactions or metastatic potential, warranting further functional investigation.

Immune profiling revealed that MACCS2 had significantly higher expression of checkpoint molecules (*CD274*, *CD247*, *PDCD1LG2*) and increased infiltration of effector immune cells, forming an “immune-hot” phenotype with greater predicted sensitivity to ICIs. CD274 and PDCD1LG2, known ligands of PD-1, contribute to immune regulation by enhancing Treg function and preventing immune dysregulation [44–46]; while CD247 is critical for T cell receptor signaling and antigen recognition [47, 48]. Enrichment analysis confirmed that MACCS2 tumors were activated in CD8⁺ T cell effector and antigen presentation pathways, both essential for anti-tumor immunity [49–51]. By contrast, MACCS1 displayed high TMB but low immune activation, and was enriched in EMT and DNA repair pathways, suggesting that it escapes immune recognition through genomic stability maintenance and immunosuppressive mechanisms [52, 53]. The findings were significantly associated with a poor prognosis and shortened survival in this molecular subtype.

Drug sensitivity profiling identified subtype-specific therapeutic vulnerabilities. MACCS1 showed higher predicted sensitivity to axitinib, pazopanib, and several small molecules targeting oncogenic pathways such as PI3K/mTOR and RAS/RAF/MEK, consistent with its proliferative phenotype. Axitinib and pazopanib, which inhibit VEGFRs, PDGFRs, FGFRs, and c-Kit, are already used clinically in renal cell carcinoma, lung cancer, and other malignancies [54–56]. By contrast, MACCS2 was more sensitive to inhibitors of EGFR, MAPK, and JAK/STAT signaling, further highlighting its dependence on signal transduction pathways [57–59]. Although these in silico drug sensitivity predictions provide valuable hypotheses, they are based on cell line data and computational modeling. Differences between in vitro models and patient tumors, including microenvironmental factors and drug metabolism, may limit direct clinical applicability. Therefore, experimental and clinical validation is essential before therapeutic application.

This study has several limitations. First, the analyses were based on publicly available datasets and a limited number of clinical samples, which may not fully capture the heterogeneity of ACC. Second, the sample size in some omics layers was relatively small, potentially affecting statistical power. Third, although in vitro experiments were performed, in vivo validation in appropriate animal models or prospective clinical cohorts is still lacking. These factors should be addressed in future work to strengthen the translational potential of the MACCS classification and its associated biomarkers.

This study also highlights *HOXC11* as a candidate diagnostic and therapeutic target. Using random forest and Cox regression analysis, *HOXC11* emerged as the most significant prognostic gene in MACCS1. High *HOXC11* expression correlated with *TP53* and *PKHD1* mutations, increased chromosomal deletions (9p23, 9p21.3), and activation of cell cycle–related pathways such as the G2/M checkpoint and E2F targets, all consistent with a proliferative drive. Functionally, *HOXC11* knockdown significantly reduced proliferation and colony formation in vitro, confirming its role in ACC tumor growth. Previous studies in lung, breast, and colorectal cancers have shown that aberrant *HOXC11* activity disrupts normal cell cycle regulation, DNA repair, and apoptosis, thereby promoting unchecked cell proliferation and tumor progression [60–62]. Our findings extend this evidence to ACC and support *HOXC11* as a biomarker of poor prognosis and a potential therapeutic target, particularly in the high-risk MACCS1 subtype.

Despite its strengths, this study has limitations. First, the analyses relied on publicly available datasets with relatively small sample sizes, which may not fully capture the heterogeneity of ACC. Second, some omics layers had limited statistical power. Third, while in vitro assays were performed, in vivo validation using animal models or clinical cohorts is still lacking. Addressing these limitations in future work will be crucial to strengthen the translational potential of the MACCS classification and its associated biomarkers.

### Summarize

This study successfully constructed a molecular classification system for ACC through multi-omics consensus analysis, which profoundly revealed the significant differences in prognosis, biological behavior, genomic landscape and treatment sensitivity among different subtypes. Most importantly, this classification directly guides the stratification of treatment strategies: patients with the MACCS2 subtype may benefit from immune checkpoint inhibitor therapy, while patients with the MACCS1 subtype are more sensitive to specific targeted drugs. In addition, the identified key factor of high-risk subtype, *HOXC11*, is not only a powerful prognostic biomarker, but also a potential therapeutic target with preliminary functional verification. These findings not only deepen our understanding of the molecular mechanisms of ACC, a difficult-to-treat disease, but also provide a solid theoretical basis for the clinical implementation of precision treatment based on molecular typing and the development of new targeted drugs, which is expected to significantly improve the clinical prognosis of ACC patients.

## Supplementary Information

Supplementary material 1.

## Data Availability

Multi-omics data of ACC patients were obtained from The Cancer Genome Atlas (TCGA, https://portal.gdc.cancer.gov/), including transcriptome profiles, DNA methylation, somatic mutations, and corresponding survival information. Only patients with complete multi-omics and prognostic data were included in the analysis. For external validation, four independent ACC gene expression datasets were retrieved from the Gene Expression Omnibus (GEO) database: GSE10927 (*n* = 24), GSE33371 (*n* = 23), GSE70621 (*n* = 29), and GSE19750 (*n* = 22). As all data were derived from public repositories, no additional ethics approval was required.

## References

[1] Ghosh C, Hu J. Advances in translational research of the rare cancer type adrenocortical carcinoma. Nat Rev Cancer. 2023;23(12):805–24. 37857840 10.1038/s41568-023-00623-0

[2] Ilanchezhian M, Varghese DG, Glod JW, et al. Pediatric adrenocortical carcinoma. Front Endocrinol (Lausanne). 2022;13:961650. 36387865 10.3389/fendo.2022.961650 PMC9659577

[3] LIBé R, Huillard O. Adrenocortical carcinoma: diagnosis, prognostic classification and treatment of localized and advanced disease. Cancer Treat Res Commun. 2023;37:100759. 37690343 10.1016/j.ctarc.2023.100759

[4] Sarrafan-Chaharsoughi Z, Yazdian Anari P, Malayeri AA, et al. Update on adrenocortical carcinoma. Urol Clin North Am. 2025;52(2):275–86. 40250894 10.1016/j.ucl.2025.01.009

[5] Puglisi S, Calabrese A, Basile V, et al. New perspectives for mitotane treatment of adrenocortical carcinoma. Best Pract Res Clin Endocrinol Metab. 2020;34(3):101415. 32179008 10.1016/j.beem.2020.101415

[6] Sun J, Huai J, Zhang W, et al. Therapeutic strategies for adrenocortical carcinoma: integrating genomic insights, molecular targeting, and immunotherapy. Front Immunol. 2025;16:1545012. 40145087 10.3389/fimmu.2025.1545012 PMC11937102

[7] Kuhlen M, Schmutz M, Kunstreich M, et al. Targeting pediatric adrenocortical carcinoma: molecular insights and emerging therapeutic strategies. Cancer Treat Rev. 2025;136:102942. 40258305 10.1016/j.ctrv.2025.102942

[8] Kimpel O, Dischinger U. Current evidence on local therapies in advanced adrenocortical carcinoma. Horm Metab Res. 2024;56(1):91–8. 38171374 10.1055/a-2209-6022 PMC10764152

[9] Sinclair TJ, Alobuia WM, Gillis A, et al. Surgery for adrenocortical carcinoma: when and how? Best Pract Res Clin Endocrinol Metab. 2020;34(3):101408. 32265101 10.1016/j.beem.2020.101408

[10] Araujo-Castro M, Pascual-Corrales E, Molina-Cerrillo J, et al. Immunotherapy in adrenocortical carcinoma: predictors of response, efficacy, safety, and mechanisms of resistance. Biomedicines. 2021;9(3):304. 33809752 10.3390/biomedicines9030304 PMC8002272

[11] Georgantzoglou N, Kokkali S, Tsourouflis G, et al. Tumor microenvironment in adrenocortical carcinoma: barrier to immunotherapy success? Cancers (Basel). 2021. 10.3390/cancers13081798. 33918733 10.3390/cancers13081798 PMC8069982

[12] Wang SJ, Dougan SK, Dougan M. Immune mechanisms of toxicity from checkpoint inhibitors. Trends Cancer. 2023;9(7):543–53. 37117135 10.1016/j.trecan.2023.04.002 PMC10330206

[13] Zhang C, Zhang C, Wang H. Immune-checkpoint inhibitor resistance in cancer treatment: current progress and future directions. Cancer Lett. 2023;562:216182. 37076040 10.1016/j.canlet.2023.216182

[14] Ronchi CL, Altieri B, Kroiss M, et al. Next-generation therapies for adrenocortical carcinoma. Best Pract Res Clin Endocrinol Metab. 2020;34(3): 101434. 32622829 10.1016/j.beem.2020.101434

[15] Hescheler DA, Hartmann MJM, Riemann B et al. Targeted therapy for adrenocortical carcinoma: A Genomic-Based search for available and emerging options. Cancers (Basel), 2022, 14(11). 10.3390/cancers14112721 PMC9179357 35681700

[16] Berruti A, Ferrero A, Sperone P, et al. Emerging drugs for adrenocortical carcinoma. Expert Opin Emerg Drugs. 2008;13(3):497–509. 18764725 10.1517/14728214.13.3.497

[17] Sukrithan V, Husain M. Emerging drugs for the treatment of adrenocortical carcinoma. Expert Opin Emerg Drugs. 2021;26(2):165–78. 33896321 10.1080/14728214.2021.1920922

[18] Cremaschi V, Abate A, Cosentini D, et al. Advances in adrenocortical carcinoma pharmacotherapy: what is the current state of the art? Expert Opin Pharmacother. 2022;23(12):1413–24. 35876101 10.1080/14656566.2022.2106128

[19] Ng L, Libertino JM. Adrenocortical carcinoma: diagnosis, evaluation and treatment. J Urol. 2003;169(1):5–11. 12478091 10.1016/S0022-5347(05)64023-2

[20] Shebrain S. Prediction of survival in adrenocortical carcinoma. J Invest Surg. 2022;35(5):1161–2. 34663167 10.1080/08941939.2021.1991057

[21] VIëTOR CL, Schurink IJ, GRüNHAGEN DJ, et al. Primary tumour resection in metastasised adrenocortical carcinoma. Endocr Relat Cancer. 2025. 10.1530/ERC-24-0056. 39652308 10.1530/ERC-24-0056

[22] Lu Q, Nie R, Luo J, et al. Identifying immune-specific subtypes of adrenocortical carcinoma based on immunogenomic profiling. Biomolecules. 2023;13(1):104. 36671489 10.3390/biom13010104 PMC9855412

[23] Cantini L, Zakeri P, Hernandez C, et al. Benchmarking joint multi-omics dimensionality reduction approaches for the study of cancer. Nat Commun. 2021;12(1):124. 33402734 10.1038/s41467-020-20430-7 PMC7785750

[24] Zheng S, Cherniack AD, Dewal N, et al. Comprehensive pan-genomic characterization of adrenocortical carcinoma. Cancer Cell. 2016;29(5):723–36. 27165744 10.1016/j.ccell.2016.04.002 PMC4864952

[25] Lu X, Meng J, Zhou Y, Jiang L, Yan F. MOVICS: an R package for multi-omics integration and visualization in cancer subtyping. Affiliations State Key Laboratory of Natural Medicines, Research Center of Biostatistics and Computational Pharmacy, China Pharmaceutical University, Nanjing 210009, China Department of Urology, The First Affiliated Hos, 2020, 36(22–23): 5539-41.

[26] Chalise P, Fridley BL. Integrative clustering of multi-level ‘omic data based on non-negative matrix factorization algorithm. PLOS ONE. 2017;12(No.5):e0176278. 28459819 10.1371/journal.pone.0176278 PMC5411077

[27] Tibshirani R, Walther G, Hastie T. Estimating the number of clusters in a data set via the gap statistic. Stanford University, USA, 2001, 63(2): 411 − 23.

[28] Love MI, Huber W, Anders S. Moderated estimation of fold change and dispersion for RNA-seq data with DESeq2(Article). Harvard School of Public Health, Department of Biostatistics and Computational Biology, Dana Farber Cancer Institute and Department of Biostatistics, 450 Brookline Avenue, Boston, MA, United States; European Molecular Biology Laboratory, G, 2014, 15(12): 550. 10.1186/s13059-014-0550-8 PMC4302049 25516281

[29] Rooney MS, Shukla SA, Wu CJ, et al. Molecular and genetic properties of tumors associated with local immune cytolytic activity. Cell. 2015;160(1–2):48–61. 25594174 10.1016/j.cell.2014.12.033 PMC4856474

[30] Xu Q, Xu H, Deng R, Wang Z, Li N, Qi Z, Zhao J, Huang W. Multi-omics analysis reveals prognostic value of tumor mutation burden in hepatocellular carcinoma. Affiliations The Second Affiliated Hospital and Yuying Children’s Hospital of Wenzhou Medical University, No 109 Xueyuan West Road, Wenzhou, 325000, Zhejiang, China Zhejiang University School of Medicine, Hangzhou, 310009, Zh, 2021, Vol.21(No.1): 342. 10.1186/s12935-021-02049-w PMC8254981 34217320

[31] Xu F, Guan Y, Zhang P, et al. Tumor mutational burden presents limiting effects on predicting the efficacy of immune checkpoint inhibitors and prognostic assessment in adrenocortical carcinoma. Department Med Xi’an Jiaotong Univ Department Med Xi’an Jiaotong Univ Department Urol Second Affiliated Hosp Xi’an Jiaotong Univ Department Urol Second Affiliated H. 2022;221:1–14.

[32] Li C, Mao Y, Liu Y, Hu J, Su C, Tan H, Hou X, Ou M. Machine learning-based integration develops a multiple programmed cell death signature for predicting the clinical outcome and drug sensitivity in colorectal cancer. Affiliations central laboratory. Second Affiliated Hosp Guilin Med Universi. 2025;36(1):1–18. Key Laboratory of Glucose and Lipid Metabolism DisordersThe Second Affiliated Hospital of Guilin Medical University Guangxi Health Commission. 10.1097/CAD.0000000000001654 39132895

[33] Lim J, Jung h D, Lee K-M et al. Genome-scale metabolic modeling reveals a metabolic switch that restores sensitivity to anticancer chemotherapy in drug-resistant breast cancer cells. Cancer Res, 2023, 83(7).

[34] LAMB J, CRAWFORD E D PECKD, et al. The connectivity map: using gene-expression signatures to connect small molecules, genes, and disease. Science. 2006;313(5795):1929–35. 17008526 10.1126/science.1132939

[35] Brandau M, Kirsch V, Chernyakov D, et al. KO of ELAVL1 effects steroid synthesis in ACC cell line NCI-H295R. Univ Clin Halle Dept Intern Med 4 Halle Germany;Univ Witten Herdecke Inst Physiol Pathophysiol Toxicol Witten Germany;Univ Clin Wurzburg Dept Intern Med 1 Wurzburg Ger. 2022;236:454.

[36] Nishi H, Arai H. NCI-H295R, a human adrenal cortex-derived cell line, expresses purinergic receptors linked to Ca²⁺-mobilization/influx and cortisol secretion. PLoS ONE. 2013;8(8):e71022. 23951072 10.1371/journal.pone.0071022 PMC3738630

[37] Gara SK, Lack J, Zhang L, et al. Metastatic adrenocortical carcinoma displays higher mutation rate and tumor heterogeneity than primary tumors. Nat Commun. 2018;9(1):4172. 30301885 10.1038/s41467-018-06366-z PMC6178360

[38] Fassnacht M, LIBé R, Kroiss M, et al. Adrenocortical carcinoma: a clinician’s update. Nat Rev Endocrinol. 2011;7(6):323–35. 21386792 10.1038/nrendo.2010.235

[39] Sasano H, Suzuki T, Moriya T. Recent advances in histopathology and immunohistochemistry of adrenocortical carcinoma. Endocr Pathol. 2006;17(4):345–54. 17525483 10.1007/s12022-006-0006-0

[40] Ali AE, Raphael SJ. Functional oncocytic adrenocortical carcinoma. Endocr Pathol. 2007;18(3):187–9. 18058268 10.1007/s12022-007-9000-4

[41] Bertherat J, Coste J, Bertagna X. Adjuvant mitotane in adrenocortical carcinoma. N Engl J Med. 2007;357(12):1256–7. 17881760 10.1056/NEJMc076267

[42] Chen X, Sandrine IK, Yang M, et al. MUC1 and MUC16: critical for immune modulation in cancer therapeutics. Frontiers in immunology. 2024;15:1356913. 38361923 10.3389/fimmu.2024.1356913 PMC10867145

[43] Lu F, Ma Q, Xie W, et al. CMYA5 establishes cardiac dyad architecture and positioning. Nat Commun. 2022;13(1):2185. 35449169 10.1038/s41467-022-29902-4 PMC9023524

[44] Alexander PG, Mcmillan DC, Park JH. A meta-analysis of CD274 (PD-L1) assessment and prognosis in colorectal cancer and its role in predicting response to anti-PD-1 therapy. Crit Rev Oncol Hematol. 2021;157:103147. 33278675 10.1016/j.critrevonc.2020.103147

[45] Dermani FK, Samadi P, Rahmani G, et al. PD-1/PD-L1 immune checkpoint: potential target for cancer therapy. J Cell Physiol. 2019;234(2):1313–25. 30191996 10.1002/jcp.27172

[46] Chen Q, Liu M, Zhao P, et al. Prognostic significance of PDCD1LG2 expression in pan-cancer and its relationship with the immune microenvironment. Asian J Surg. 2024;47(12):5288–90. 38944602 10.1016/j.asjsur.2024.06.016

[47] Aguinaga-Barrilero A, Castro-Sánchez P, Juárez I, et al. Defects at the Posttranscriptional Level Account for the Low TCRζ Chain Expression Detected in Gastric Cancer Independently of Caspase-3 Activity. J Immunol Res. 2020;2020:1039458. 33354577 10.1155/2020/1039458 PMC7737443

[48] Ferrandina G, Ranelletti FO, Legge F, et al. Celecoxib up-regulates the expression of the zeta chain of T cell receptor complex in tumor-infiltrating lymphocytes in human cervical cancer. Clin Cancer Res. 2006;12(7 Pt 1):2055–60. 16609015 10.1158/1078-0432.CCR-05-2530

[49] Huang L, Wang D, Xu M, et al. Mixed radiation with different doses induces CCL17 to recruit CD8(+)T cell to exert anti-tumor effects in non-small cell lung cancer. Front Immunol. 2024;15:1508007. 39877375 10.3389/fimmu.2024.1508007 PMC11772420

[50] Wang Q, Qin Y, Li B. CD8(+) T cell exhaustion and cancer immunotherapy. Cancer Lett. 2023;559:216043. 36584935 10.1016/j.canlet.2022.216043

[51] Yang K, Halima A, Chan TA. Antigen presentation in cancer - mechanisms and clinical implications for immunotherapy. Nat Rev Clin Oncol. 2023;20(9):604–23. 37328642 10.1038/s41571-023-00789-4

[52] Nachiyappan A, Gupta N. EHMT1/EHMT2 in EMT, cancer stemness and drug resistance: emerging evidence and mechanisms. FEBS J. 2022;289(5):1329–51. 34954891 10.1111/febs.16334

[53] Chalmers ZR, Connelly CF. Analysis of 100,000 human cancer genomes reveals the landscape of tumor mutational burden. Genome Med. 2017;9(1):34. 28420421 10.1186/s13073-017-0424-2 PMC5395719

[54] Ferrarotto R, Sousa LG, Feng L, et al. Phase II clinical trial of axitinib and avelumab in patients with recurrent/metastatic adenoid cystic carcinoma. J Clin Oncol. 2023;41(15):2843–51. 36898078 10.1200/JCO.22.02221 PMC10414730

[55] Plimack ER, Powles T. Pembrolizumab plus axitinib versus sunitinib as first-line treatment of advanced renal cell carcinoma: 43-month follow-up of the phase 3 KEYNOTE-426 study. Eur Urol. 2023;84(5):449–54. 37500340 10.1016/j.eururo.2023.06.006

[56] Rini BI, Plimack ER, Stus V, et al. Pembrolizumab plus axitinib versus Sunitinib for advanced Renal-Cell carcinoma. N Engl J Med. 2019;380(12):1116–27. 30779529 10.1056/NEJMoa1816714

[57] Nussinov R, Yavuz BR, Jang H. Anticancer drugs: how to select small molecule combinations? Trends Pharmacol Sci. 2024;45(6):503–19. 38782689 10.1016/j.tips.2024.04.012 PMC11162304

[58] Wu Q, Qian W, Sun X, et al. Small-molecule inhibitors, immune checkpoint inhibitors, and more: FDA-approved novel therapeutic drugs for solid tumors from 1991 to 2021. J Hematol Oncol. 2022;15(1):143. 36209184 10.1186/s13045-022-01362-9 PMC9548212

[59] Wang P, Jin X. Recent advances in small molecule prodrugs for cancer therapy. Anticancer Agents Med Chem. 2014;14(3):418–39. 23869777 10.2174/18715206113139990317

[60] Han L, Gong F, Wu X, et al. Comprehensive characterization of PKHD1 mutation in human colon cancer. Cancer Med. 2024;13(1):e6796. 38178618 10.1002/cam4.6796 PMC10807659

[61] Shang T, Chen X, Xue H, et al. The *PKHD1* gene inhibits tumor proliferation and invasion in intrahepatic cholangiocarcinoma by activating the Notch pathway. Int J Med Sci. 2024;21(14):2655–63. 39512694 10.7150/ijms.95964 PMC11539381

[62] Giacomelli AO, Yang X, Lintner RE, et al. Mutational processes shape the landscape of TP53 mutations in human cancer. Nat Genet. 2018;50(10):1381–7. 30224644 10.1038/s41588-018-0204-y PMC6168352

