# Supplementary figures and images for: Multi-omics-based molecular classification of adrenocortical carcinoma predicts response to immunotherapy and targeted treatments
Source: Discov Oncol. 2025 Oct 2;16:1803. doi: 10.1007/s12672-025-03649-y (PMC12491137; doi:10.1007/s12672-025-03649-y)

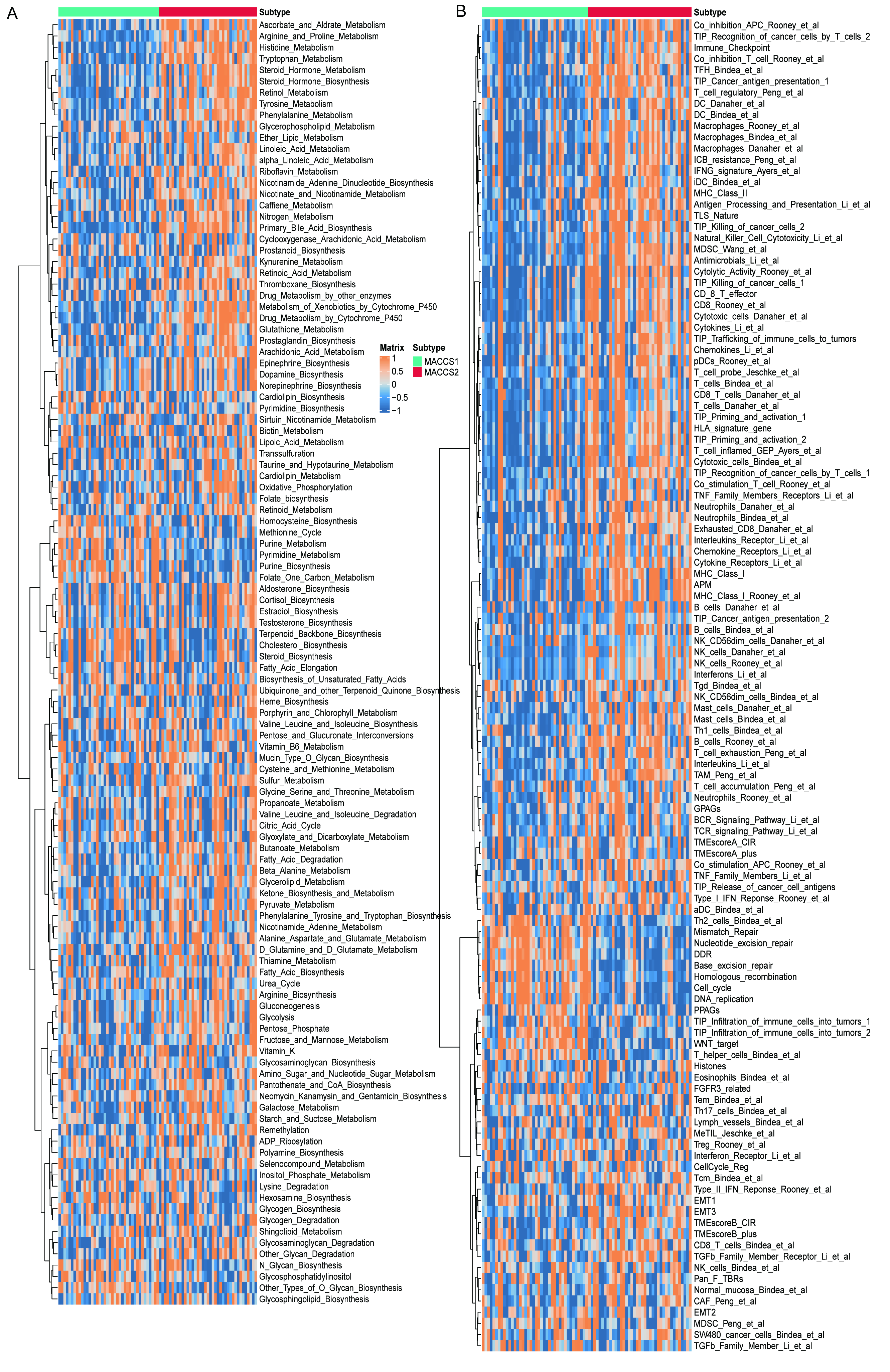

Supplement: Supplementary file 1 — Supplementary material 1. [file 12672_2025_3649_MOESM1_ESM.tif]
